# HIV infection, viral hepatitis and liver fibrosis among prison inmates in West Africa

**DOI:** 10.1186/s12879-016-1601-4

**Published:** 2016-06-06

**Authors:** Antoine Jaquet, Gilles Wandeler, Judicaël Tine, Claver A. Dagnra, Alain Attia, Akouda Patassi, Abdoulaye Ndiaye, Victor de Ledinghen, Didier K. Ekouevi, Moussa Seydi, François Dabis

**Affiliations:** Université Bordeaux, ISPED, Centre INSERM U897- Epidémiologie-Biostatistique, F-33000 Bordeaux, France; INSERM, ISPED, Centre INSERM U897- Epidémiologie-Biostatistique, F-33000 Bordeaux, France; Service de maladies infectieuses et tropicales, CRCF, CHU de Fann, Dakar, Sénégal; Department of Infectious Diseases, University Hospital Bern, Bern, Switzerland; Institute of Social and Preventive Medicine, University of Bern, Bern, Switzerland; Service de virologie, BIOLIM, Université de Lomé, Lomé, Togo; Service de hépato-gastroentérologie, CHU de Yopougon, Abidjan, Côte d’Ivoire; Service de maladies infectieuses et tropicales, CHU Sylvanus Olympio, Lomé, Togo; Pavillon Spécial, Hôpital Aristide Le Dantec, Dakar, Sénégal; Centre d’investigation de la fibrose hepatique, Hopital Haut-Leveque, CHU de Bordeaux & INSERM U1053, Université de Bordeaux, Bordeaux, France; Département de Santé Publique, Faculté des Sciences de la santé, Université de Lomé, Lomé, Togo

**Keywords:** Liver fibrosis, HIV, Hepatitis B, Hepatitis C, Africa

## Abstract

**Background:**

Prisoners represent a vulnerable population for blood-borne and sexually transmitted infections which can potentially lead to liver fibrosis and ultimately cirrhosis. However, little is known about the prevalence of liver fibrosis and associated risk factors among inmates in sub-Saharan Africa.

**Methods:**

Screening of liver fibrosis was undertaken in a randomly selected sample of male inmates incarcerated in Lome, Togo and in Dakar, Senegal using transient elastography. A liver stiffness measurement ≥9.5 KPa was retained to define the presence of a severe liver fibrosis. All included inmates were also screened for HIV, Hepatitis B Virus (HBV) and Hepatitis C Virus (HCV) infection. Substances abuse including alcohol, tobacco and cannabis use were assessed during face-to-face interviews. Odds Ratio (OR) estimates were computed with their 95 % Confidence Interval (CI) to identify factors associated with severe liver fibrosis.

**Results:**

Overall, 680 inmates were included with a median age of 30 years [interquartile range: 24–35]. The prevalence of severe fibrosis was 3.1 % (4.9 % in Lome and 1.2 % in Dakar). Infections with HIV, HBV and HCV were identified in 2.6 %, 12.5 % and 0.5 % of inmates, respectively. Factors associated with a severe liver fibrosis were HIV infection (OR = 7.6; CI 1.8–32.1), HBV infection (OR = 4.8; CI 1.8–12.8), HCV infection (OR = 52.6; CI 4.1–673.8), use of traditional medicines (OR = 3.7; CI 1.4–10.1) and being incarcerated in Lome (OR = 3.3; CI 1.1–9.8) compared to Dakar.

**Conclusions:**

HIV infection and viral hepatitis infections were identified as important and independent determinants of severe liver fibrosis. While access to active antiviral therapies against HIV and viral hepatitis expands in Africa, adapted strategies for the monitoring of liver disease need to be explored, especially in vulnerable populations such as inmates.

## Background

Worldwide, liver cirrhosis, the final stage of liver fibrosis, is a significant public health problem. Indeed, deaths attributable to liver cirrhosis increased from around 676,000 in 1980 to over 1 million in 2010 representing about 2 % of the overall number of deaths [1]. However, the paucity of data on the specific causes of death in sub-Saharan Africa severely limits a reliable assessment of the true burden of disease in this part of the world [2]. Diagnosis of liver fibrosis classically relies on histology through liver biopsy. However, this invasive method has many limitations including iatrogenic effects, high cost and the need for adequately trained pathologists, challenging its use in low-income countries. Recently, several non-invasive liver fibrosis measurement methods have been developed to overcome some of the challenges related to liver biopsy. Of these, transient elastography (Fibroscan®, Echosens, France) based on liver stiffness measurement (LSM), has proven to be accurate in a wide spectrum of liver diseases including hepatitis C virus (HCV) and hepatitis B virus (HBV) infections [3–6]. The availability of these simple diagnostic tools as well as treatment options for many of these etiologic conditions makes the assessment of liver fibrosis particularly relevant, especially in populations presenting a high risk of liver disease [7].

Prisons are known to be a high-risk environment for addictive behaviors as well as blood-borne and sexually transmitted infections which can lead to liver damage [8]. Several factors contribute to this increased risk of communicable diseases including promiscuity, risk behaviors as well as poor access to diagnostic and preventive measures. Indeed, infection with Human Immunodeficiency Virus (HIV) as well as chronic HBV and HCV infections are common in these populations [9, 10]. In sub-Saharan Africa where few data on the health of prisoners are available, studies have mainly focused on the prevalence of HIV infection and tuberculosis [11–15]. However, little is known about the prevalence of viral hepatitis in these settings. In West Africa, two studies conducted in Nigeria and Ghana a few years ago showed a particularly high prevalence of viral hepatitis in prisons [16–18].

In a context of expanding access to efficient antiviral therapies for HIV infection and viral hepatitis in sub-Saharan Africa, there is a need to document the level liver damage in vulnerable populations such as inmates. Our aim was to investigate liver fibrosis and associated factors among inmates incarcerated in West Africa.

## Methods

### Study population and data collection

Screening of liver fibrosis was undertaken in a randomly selected sample of inmates incarcerated in September - October 2013 in the state prison of Lome (Togo) and in April - May 2014 in the state prison of Dakar (Senegal). For logistics reasons and acceptability, we conducted a cluster sampling approach using prison cells as the statistical units. Prison cells were mapped and ranked according to their size (number of prisoners currently incarcerated in each cell during the study period). A randomly selected group of cells were then selected by applying sampling interval to this list in order to identify a study sample of approximately 350 inmates in each participating state prison. All the inmates from a selected cell were invited to participate. Only adults men (≥18 years) were included. All included inmates were informed about implications related to their study participation and provided their signed informed consent. All participants accessed to a baseline medical consultation and received counseling with regards to their serological status if positive for HIV and/or hepatitis viruses. The national programs for HIV/AIDS in Senegal and Togo currently ensure a universal access to care and treatment with highly active antiretroviral for eligible patients. Both prisons have a medical unit that ensures access to HIV care supported by the national HIV/AIDS program. All participants with a positive HIV test were notified to the national HIV program of their respective country and accessed to HIV care and treatment, if eligible. As access to treatment for hepatitis B or C is currently not free of charge, we were not able to guarantee treatments and long term follow-up of inmates who had a positive hepatitis B or C screening test. Prison doctors were in charge to reefer participants with a positive HBV or HCV test to infectious disease units in teaching hospitals in Lome (Silvanus Olympio university hospital) and Dakar (Fann university hospital) for a free baseline consultation once all biological tests including viral loads were available.

Standardized questionnaires were administered in French or local language by nurses from the prison medical staff through face-to-face interviews. Socio-demographic data (age, number and duration of incarcerations) as well as behavioral information including drug use (alcohol, tobacco, cannabis, cocaine/crack and heroin) were reported. Alcohol use during the last 12 months was scored with the alcohol use disorder identification test (AUDIT). This test developed by the World Health Organization (WHO) has been specifically designed to screen for individuals experiencing active alcohol abuse and/or dependence. The AUDIT includes 10 questions, each of them scored from 0 to 4 points for a maximum score of 40 points. According to their AUDIT score patients were classified as abstinent (AUDIT = 0), present alcohol users or hazardous drinkers using a standard cut-off of ≥8 points [19]. Self-report of tobacco, cannabis, cocaine/crack and heroin use allowed the classification of the respondents as nonusers, past users or current users. Information on present or past history of injection drug use was also collected. The ingestion of herbs through traditional medicine was reported based on participants declaration categorized as nonusers, occasional users or frequent users. Height and weight were systematically measured and reported on the case report form in order to compute the body mass index (BMI).

### Laboratory measurements

Viral hepatitis infections were assessed using rapid diagnostic tests: Determine® (Alere, Waltham, MA, United States of America (USA)) for HBs antigen and Oraquick® (Orasure, Bethlehem, PA, USA) for anti-HCV antibodies as these tests have shown relatively good diagnostic accuracy [20–22]. Infection with HIV was first assessed with the rapid diagnostic test Determine® (Alere, Waltham, MA, USA) and confirmed with additional tests according to national recommended algorithms in Senegal and Togo. Alanine aminotransferase (ALT) and Aspartate aminotransferase (AST) were also measured for participants who were positive for HBV and/or HCV rapid diagnostic tests. Results of ALT/AST were reported in international units (IU) per L. For ALT a threshold of 30 IU/L was considered to define ALT elevation [23]. Those who were positive for the HBV test underwent a viral load quantification by polymerase chain reaction using the COBAS(R) AmpliPrep/COBAS(R) TaqMan(R) v2.0 (Roche®) kit in Senegal and the ABBOTT M2000RT (Abbot®) kit in Togo. Inmates from Lome and Dakar with a positive HCV test underwent viral load quantification by polymerase chain reaction using the Roche® kit centralized in Dakar. Results were reported in international units (IU) per ml. For HBV, a threshold of 2 000 IU/ml was considered for clinically significant viral load replication and a threshold of 20 000 IU/ml for high viral load replication as suggested in the recent European guidelines for the management of chronic HBV infection [24]. All laboratory tests were performed according to the manufacturer’s specifications.

### Transient elastography

All participants were assessed for liver fibrosis using a portable transient elastography device (Fibroscan 402®, Echosens, Paris) with an M probe. A single device was made available for a two-month period in each participating state prison. A maximum of two operators were specifically trained to perform the examination at each site. All operators followed the online formal training and certification from the manufacturer. Prior to the study inclusions, they all went through a practical training session supervised by experienced physicians. To be considered reliable, the examination must include at least 10 measurements with an interquartile range (IQR) equal or below 30 % of the median value (IQR/LSM ≤30 %) [25]. Participants for who these criteria were not achieved after several attempts were excluded from the present analysis. The median value of ten successive validated LSM was used to represent liver stiffness.

### Statistical analysis

A cutoff value of LSM ≥9.5 kPa was used to define the presence of severe liver fibrosis. This threshold was reported in a recent meta-analysis as the median optimal cut-off to define a severe liver fibrosis that approximates a ≥ F3 stage using the METAVIR score among populations presenting with various etiological factors for liver fibrosis. A cut-off value of ≥14.4 KPa was used to define the presence of cirrhosis [26].

Pearson’s *χ*^2^ test or Fisher’s exact test were used to compare categorical variables according to the presence of a severe liver fibrosis or to participating state prisons. Kruskall-Wallis test was used for comparisons between continuous variables. A corrected logistic regression using the Firth’s penalized likelihood method was used to assess factors associated with severe liver fibrosis [27]. A stepwise descending procedure was used to select the final multivariable model. All potential confounding variables were included in the initial model. The Akaike information criterion was used to assess the goodness of fit of the model. A low value of this criterion is associated with a better prediction of the model. Proportions and Odds Ratio (OR) estimates were reported with their 95 % Confidence Interval (95 % CI). Statistical analyses were computed using SAS software 9.2 (SAS Institute Inc. NC. USA).

### Ethics statement

The present study is in compliance with the Helsinki Declaration and was approved by the national ethic committees of Senegal (‘Comite National d’Ethique pour la Recherche en Sante au Senegal’, approval number: 3226/MJ/DAP/SMS) and Togo (‘Comite de Bioethique pour la Recherche en Sante du Togo’, approval number: 004/2013).

## Results

Initially, 787 participants were approached to participate and 78 were excluded for the following reasons: refusals (*n* = 48), transferred to another prison (*n* = 10) or liberated (*n* = 20). Of the 709 participants screened for HIV, HBV, HCV and liver fibrosis, 29 (4.1 %) were subsequently excluded from the final analysis for the following reasons: unreliable LSM (*n* = 20), indeterminate/unknown HBV, HCV or HIV status (*n* = 4) and age < 18 years (*n* = 5). A total of 680 inmates were finally included in the analysis in Lome (*n* = 347) and Dakar (*n* = 333). Their median age was 30 years [interquartile range (IQR): 24–35] and their median time of incarceration was 8 months [IQR 2–27]. Of these inmates currently incarcerated, 125 (18.4 %) had a past history of previous incarceration. Hazardous drinking and current tobacco use were reported in 58 (8.6 %) and 315 (46.4 %) inmates, respectively. A current or past use of cannabis, cocaine/crack and intravenous use of heroin was reported in 230 (33.8 %), seven (1.0 %) and four (0.6 %) inmates, respectively.

The prevalence of severe fibrosis was 3.1 % [95 % CI 1.8–4.4] (Table 1). A marked difference in severe liver fibrosis was observed between inmates in Lome and Dakar with prevalence of 4.9 % [95 % CI 2.6–7.2] and 1.2 % [95 % CI 0.3–2.4], respectively (*p* < 0.01). No cases of cirrhosis were reported. Overall, HIV infection was identified in 2.6 % [95 % CI 1.4–3.8] of inmates, 3.5 % [95 % CI 1.5–5.4] in Lome and 1.8 % [95 % CI 0.4–3.2] in Dakar (Table 2). A past history of HIV testing prior to the study was reported by 213 (31.3 %) participants. Of the 18 inmates identified as HIV-infected, only one was aware of his HIV infection and was already on antiretroviral therapy.

**Table 1:** Main characteristics of inmates according to their place of incarceration (*n* = 680)

	State prison of Dakar (Senegal)	State prison of Lome (Togo)	p	Total
	(*n* = 333)	(*n* = 347)		(*n* = 680)
Age (median, [IQR]), years	31 [26–36]	28 [23–33]	<0.0001	30 [24–35]
Duration of incarceration (median, [IQR]), months	12 [1–34]	8 [3–18]	0.1	8 [2–27]
Past history of incarceration, n (%)			<0.0001	
No	247 (74.2)	308 (88.8)		555 (81.6)
Yes	86 (25.8)	39 (11.2)		125 (18.4)
Tobacco, n (%)			<0.0001	
No use	83 (24.7)	189 (54.5)		272 (39.9)
Past use	37 (11.1)	56 (16.1)		93 (13.7)
Present use	213 (64.2)	102 (29.4)		315 (46.4)
Cannabis, n (%)			<0.0001	
No use	180 (54.1)	270 (77.8)		450 (66.2)
Past use	128 (38.4)	51 (14.7)		179 (26.3)
Present use	25 (7.5)	26 (7.5)		51 (7.5)
Alcohol use^a^, n (%)			<0.0001	
No	305 (91.6)	222 (63.7)		526 (77.4)
Current drinking	10 (3.0)	86 (24.8)		96 (14.1)
Hazardous drinking	18 (5.4)	40 (11.5)		58 (8.5)
Cocaine/crack, n (%)			0.43	
No use	329 (98.8)	344 (99.1)		673 (99.0)
Present/Past use	4 (1.2)	3 (0.9)		7 (1.0)
Heroin^b^, n (%)			0.97	
No use	331 (99.4)	345 (99.4)		676 (99.4)
Present/Past use	2 (0.6)	2 (0.6)		4 (0.6)
HIV test^c^, n (%)			0.18	
Negative	327 (98.2)	335 (96.5)		662 (97.4)
Positive	6 (1.8)	12 (3.5)		18 (2.6)
HBV test^c^, n (%)			0.21	
Negative	286 (85.9)	309 (89.0)		595 (87.5)
Positive	47 (14.1)	38 (10.9)		85 (12.5)
HCV test^d^, n (%)			0.54	
Negative	331 (99.4)	346 (99.7)		677 (99.5)
Positive	2 (0.6)	1 (0.3)		3 (0.5)

^a^Reported alcohol use during the last 12 month assessed with the Alcohol Use Disorders Identification Test (AUDIT)

^b^Intravenous drug use

^c^Rapid diagnostic test: Determine® (Alere, Waltham, MA, USA)

^d^Rapid diagnostic test: Oraquick® (Orasure, Bethlehem, PA, USA)

*Abbreviations*: *HIV* Human Immunodeficiency Virus, *HBV* Hepatitis B Virus, *HCV*: Hepatitis C virus, *IQR*: Inter Quartile Range

**Table 2:** Main characteristics of inmates according to the presence of a severe liver fibrosis assessed by transient elastography (*n* = 680), 2013–2014

Median transient elastography measures	<9.5 KPa	≥9.5 KPa	p	Total
	No severe liver fibrosis	Severe liver fibrosis		
	(*n* = 659)	(*n* = 21)		(*n* = 680)
Age (median, [IQR])	30 [24–35]	31 [28–35]	0.46	30 [24–35]
State prison, n (%)			<0.01	
Dakar (Senegal)	329 (49.9)	4 (19.0)		333 (49.0)
Lome (Togo)	328 (50.1)	17 (81.0)		347 (51.0)
Tobacco, n (%)			0.98	
No use	264 (40.0)	8 (38.1)		272 (39.9)
Past use	90 (13.7)	3 (14.3)		93 (13.7)
Present use	305 (46.3)	10 (47.6)		315 (46.4)
Cannabis, n (%)			0.41	
No use	436 (66.1)	14 (66.7)		450 (66.2)
Past use	175 (26.6)	4 (19.0)		179 (26.3)
Present use	48 (7.3)	3 (14.3)		51 (7.5)
Alcohol use^a^, n (%)			0.19	
No	510 (77.4)	16 (76.2)		526 (77.4)
Current drinking	91 (13.8)	5 (23.8)		96 (14.1)
Hazardous drinking	58 (8.8)	0 (0.0)		58 (8.5)
Herbal medicine^b^, n (%)			<0.01	
No use	194 (29.4)	4 (19.0)		198 (29.1)
Occasional use	409 (62.1)	10 (47.6)		419 (61.6)
Frequent use	56 (8.5)	7 (30.4)		63 (9.3)
HIV test^c^, n (%)			0.01	
Negative	644 (97.7)	18 (85.7)		662 (97.4)
Positive	15 (2.3)	3 (14.3)		18 (2.6)
HBV test^c^, n (%)			<0.01	
Negative	581 (88.2)	14 (66.7)		595 (87.5)
Positive	78 (11.8)	7 (33.3)		85 (12.5)
HCV test^d^, n (%)			<0.01	
Negative	657 (99.7)	20 (95.2)		677 (99.5)
Positive	2 (0.3)	1 (4.8)		3 (0.5)
Body Mass Index^e^	22.8 [21.0–25.3]	22.4 `[19.8–24.4]	0.38	22.8 [21.0–25.3]

^a^Reported alcohol use during the last 12 month assessed with the Alcohol Use Disorders Identification Test (AUDIT)

^b^Reported use of traditional medicine with ingested herbs

^c^Rapid diagnostic test: Determine® (Alere, Waltham, MA, USA)

^d^Rapid diagnostic test: Oraquick® (Orasure, Bethlehem, PA, USA)

^e^47.4 % of missing values

*Abbreviations*: *HIV* Human Immunodeficiency Virus, *HBV* Hepatitis B Virus, *HCV* Hepatitis C virus, *IQR* Inter Quartile Range

**Fig. 1:**
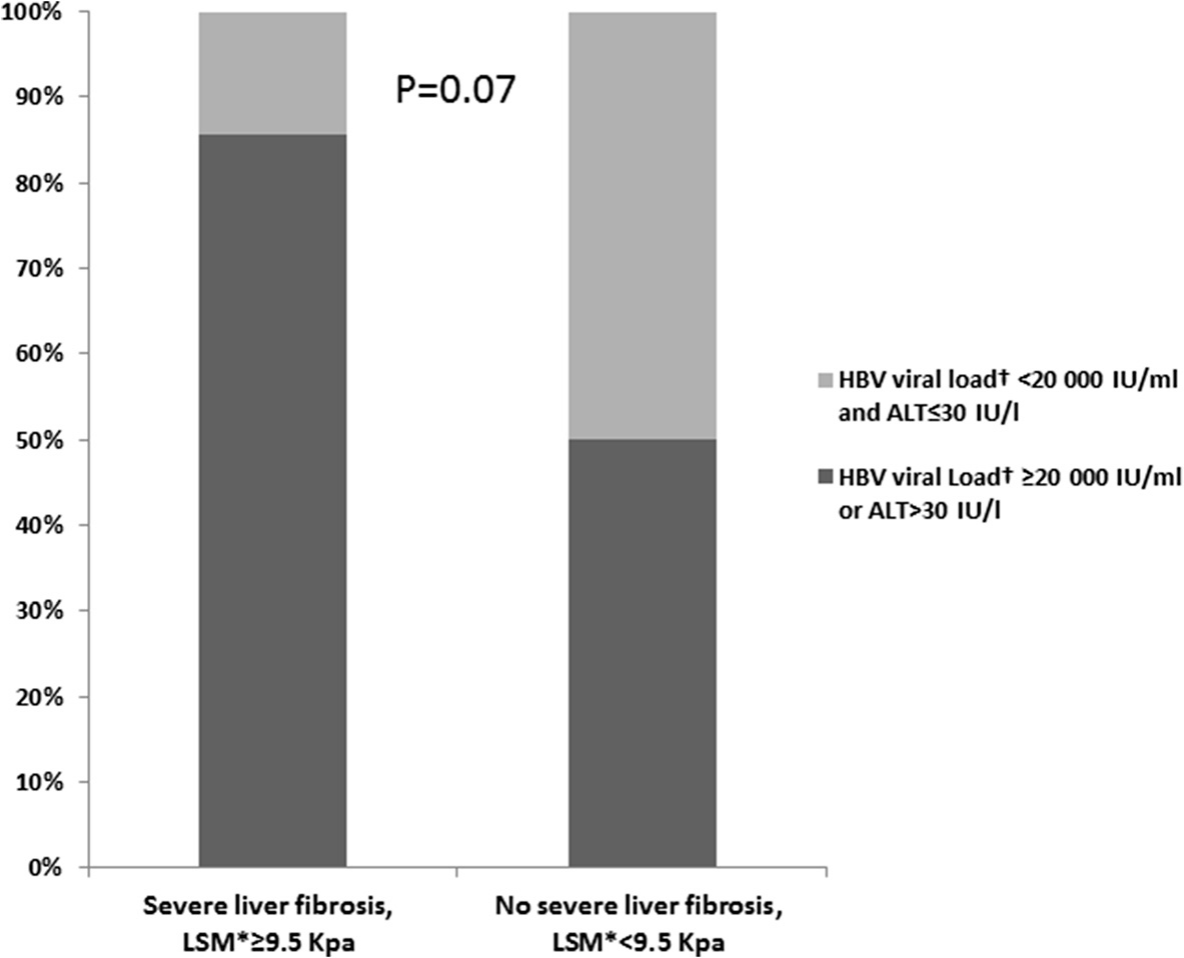
Proportion of participants with high hepatitis B Virus replication (≥20 000 IU/ml) or high ALT level (>30 IU/l) in HBV-infected inmates according to the presence of a severe liver fibrosis (*n* = 69). Lome (Togo) and Dakar (Senegal), 2013–2014. * Median liver stiffness measure. † 69 HBVviral load available out of the 85 HBV-infected participants

Infection with HBV was documented in 85 inmates (12.5 % [95 CI 10.0–15.0]) with no significant difference between inmates in Lome (10.9 %) and Dakar (14.1 %) (*p* = 0.21). Of the 85 HBV-infected inmates, seven (8.2 %) had a severe fibrosis. One patient was co-infected with both HIV and HBV. The presence of an ALT value >30 IU/ml was reported in 29 (35.8 %) HBV-positive patients, with no significant difference between participants with severe fibrosis (42.9 %) or without (35.1 %) (*P* = 0.89). The quantification of HBV viral load was performed in 69 (81.2 %) of these individuals. The remaining 16 inmates could not be assessed for HBV viral load for various reasons (refusals, released before the time of blood collection). Significant (>2 000 IU/ml) and high HBV replication levels (>20 000 IU/ml) were reported in 33 (47.8 %) and 26 (37.7 %) inmates assessed for HBV viral load, respectively. Among the seven patients with severe fibrosis, six (85.7 %) presented with a high HBV replication level or a high ALT value compared to 31 (50 %) in the remaining 62 patients without severe fibrosis (*p* = 0.07) (Fig. 1).

HCV infection was documented in three participants providing an estimated prevalence of 0.5 % [95 % CI 0.1–0.9]. Only one out of the three positive tests was confirmed by viral load quantification with a value of 5.91 log (IU/ml). None of the three prisoners positively screened for HCV infection reported intravenous drug use.

In a multivariate analysis, factors associated with a severe liver fibrosis were HIV infection (OR = 7.6; 95 % CI 1.8–32.1), HBV infection (OR = 4.8; 95 % CI 1.8–12.8), HCV infection (OR = 52.6; 95 % CI 4.1–673.8), use of traditional medicines (OR = 3.7; 95 % CI 1.4–10.1) and incarceration in Lome (OR = 3.3; 95 % CI 1.1–9.8) (reference: civil prison of Dakar) (Table 3). No significant differences in addictive behaviors according to the presence of severe liver fibrosis were noted including alcohol use (OR = 1.0; 95 % CI 0.4–3.0) versus no use or present/past cannabis use (OR = 1.0; 95 % CI 0.4–2.5) versus no use. None of the four inmates reporting intravenous drus use presented with a severe liver fibrosis.

**Table 3:** Factors associated with a severe liver fibrosis defined as a median liver stiffness measure ≥9.5 KPa in inmates of state prisons in Dakar and Lomé, West Africa (*n* = 680), 2013–2014

	Univariate analysis			Multivariable analysis	
	n/N	OR (95 % CI)	p	OR (95 % CI)	p
Age			0.81		
≤35 years	17/527	1			
>35 years	4/153	0.9 (0.3–2.5)			
State prison			0.01		0.03
Dakar, Senegal	4/333	1		1	
Lome, Togo	17/347	3.9 (1.4–11.1)		3.3 (1.1–9.8)	
Alcohol use			0.80		
No use	16/526	1			
Current/Hazardous drinking^a^	5/154	1.1 (0.4–3.0)			
HIV test^b^			<0.01		0.01
Negative	18/662	1		1	
Positive	3/18	7.9 (2.2–28.2)		7.6 (1.8–32.1)	
HBV test^b^			<0.01		<0.01
Negative	14/595	1		1	
Positive	7/85	3.8 (1.5–9.6)		4.8 (1.8–12.8)	
HCV test^c^			0.01		<0.01
Negative	20/677	1		1	
Positive	1/3	19.8(1.2–207.5)		52.6 (4.1–673.8)	
Herbal medicine^d^			<0.00		0.01
No or occasional use	14/617	1	1	1	
Frequent use	7/63	5.5 (2.2–14.0)		3.7 (1.4–10.1)	

n/N: number of inmates with sever fibrosis/total number of inmates for a specific category

^a^Reported alcohol use during the last 12 month measured with AUDIT questionnaire

^b^Rapid diagnostic test: Determine® (Alere, Waltham, MA, USA)

^c^Rapid diagnostic test: Oraquick® (Orasure, Bethlehem, PA, USA)

^d^Reported use of traditional medicine with ingested herbs

*Abbreviations*: *HIV* Human Immunodeficiency Syndrome, *HBV* Hepatitis B Virus, *HCV* Hepatitis C Virus, *OR* Odd Ratio, *CI* Confidence Interval

## Discussion

The prevalence of HIV infection was relatively high in inmates currently incarcerated in the state prisons of Lome and Dakar and independently associated with liver fibrosis. Indeed, reported HIV prevalence from these state prisons, were two to four times higher than the respective prevalence of HIV infection in the general adult population of Togo and Senegal [28]. Although sparse data exist on HIV prevalence in prisoners in these countries, our results are in accordance to a previous report from Togo where HIV prevalence in incarcerated men was estimated at 4.0 % [29]. Together with the fact that only one out of the 18 patients identified as HIV-infected was aware of its status; these results emphasize the need to promote HIV screening and access to care in prisons in sub-Saharan Africa.

The independent association between HIV infection and liver fibrosis has already been reported in sub-Saharan Africa. Stabinski *et al* conducted a case-control study on the association between HIV infection and severe liver fibrosis using the same non-invasive approach in Uganda [30]. Adjusted for various risk factors including HBV infection, HIV infection was significantly associated with liver fibrosis (OR = 1.5 [95 % CI 1.1–2.1]). Potential reasons for this association are numerous; various unmeasured tropical hepatotoxic infectious agents such as schistosomiasis may have synergistically interacted with HIV infection in the development of liver fibrosis. Our population of HIV-infected patients was mostly unaware of their HIV status and potentially had uncontrolled HIV replication. The direct toxicity of HIV on liver cells has been reported in previous studies through several mechanisms including, stellate cells activation, apoptotic effect on hepatocytes and immune activation [31–34]. A recent cohort study exploring factors associated with the occurrence of liver fibrosis in more than 14 000 HIV-infected persons identified HIV viral load as a main and independent determinant of progression to liver fibrosis [35]. Therefore, the high prevalence of liver fibrosis among HIV-infected persons reported in Uganda, Nigeria and in our study using the same methodological approach might be partly mediated by the direct toxicity of HIV itself [36]. Further etiologic and prospective studies will need to explore the exact mechanisms leading to liver fibrosis in HIV-infected persons without HBV or HCV in sub-Saharan Africa.

Both HBV and HCV infections were independently associated with severe liver fibrosis. The high prevalence of HBV infection was consistent with the prevalence of HBs antigen positivity in the general population in West Africa [37]. In addition, we provide here one of the rare reports of the impact of HBV infection on the liver. Almost 10 % of inmates positively screened for HBV were diagnosed with severe liver fibrosis making them potentially eligible to antiviral treatment regardless of other biomarkers. More than one-third of HBV-infected inmates harbored a high HBV DNA replication (>20 000 UI/ml), a well-known negative prognostic factor in HBV infection, regardless of the presence or absence of severe liver fibrosis. In a context of limited access to antiviral treatment for HBV-infected persons living in sub-Saharan Africa, the availability of reliable and appropriate tools for the assessment of liver fibrosis is of paramount importance to identify and prioritize patients in need for treatment.

A low prevalence of HCV infection was observed in our study sample, even lower than the available regional estimates [2.8 % (95%CI 2.4–3.3)] from West Africa [38]. However, this previous estimates from West Africa relied on a limited number of studies with selected populations and no information was available for several countries including Togo [39]. Prevalence studies among blood donors in Senegal reported prevalence of positive HCV serology of 0.7 % and 0.5 % [40, 41]. Therefore, the prevalence of HCV infection in West Africa is probably not homogenous according to countries and deserves more well conducted representative prevalence studies. As HCV prevalence increases with age, the fact that half of our population was under 30 might partly explain this low HCV prevalence. However, previous surveys among incarcerated people in Ghana and Nigeria have found particularly high HCV prevalence (over 10 %) [17, 18]. The spread of the HCV epidemic is mainly driven by blood exposure including intravenous drug use and unsafe medical practices through none adapted or inexistent sterilization process. In light of the marginal reporting of injected drug-use in our study sample, HCV was unlikely to have spread through this mode. Accurate estimates of HCV infection are currently limited by the availability of reliable serological assays and would benefit from larger standardized prevalence studies. Indeed, previous reports have highlighted the high rates of false positive results using the classical immunoassay techniques for the screening of HCV infection [42, 43]. Thus, the good sensitivity and specificity of the Oraquick® test used in our study might partly explain the discrepancy between our findings and previous reports on HCV prevalence in West Africa [20, 21]. While new potent but expensive cure strategies are emerging for HCV infection, a better knowledge of the epidemiology of HCV infection is urgently needed in low-income countries.

Use of herbal medicines was not uncommon in our study population and was associated with severe liver fibrosis. This finding confirmed the result from a report in Uganda where an in-depth assessment of herbs consumed showed that the Asteraceae family was associated with liver fibrosis [44]. There is a need to explore the types of herbs in relation to liver fibrosis in order to provide herb safety recommendations in the context of traditional medicine use in West Africa. Alcohol use was not associated with liver fibrosis in our report. The role of alcohol use in the development of liver fibrosis has been extensively demonstrated in high-income settings. The assessment of alcohol exposure through the AUDIT score did not capture the lifetime exposure to alcohol use which might be more relevant as liver fibrosis is a relatively long process. After adjusting for all important confounders, a fraction of the observed geographical variation in the prevalence of liver fibrosis remains unexplained. Unmeasured exposures to environmental risk factors could have played a role. For example, aflatoxin exposure which has been already linked to liver fibrosis might not be equally distributed throughout West Africa [45]. Additional investigations should explore the role of environmental factors in the occurrence of liver fibrosis and ultimately cirrhosis.

Our study population might not be representative of all prisons in these two countries as we only focused on selected civil prisons from the two main urban areas in Togo and Senegal. Indeed, the diversity of prison facilities needs to be addressed as wide variations in the prevalence of viral hepatitis infections have been reported elsewhere. Aside tobacco use, other addictive behaviors were prohibited in these prison facilities. This might have underestimated the self-reported use of drugs and alcohol. Although transient elastography is a technique rapidly mastered and easy to reproduce, it remains an operator-dependent procedure. An inadequate use of the device can lead to the overestimation of LSM. In order to provide better estimates of the burden of liver disease, larger studies with standardized procedures are needed throughout the African continent. In its latest guideline for the prevention, care and treatment of HBV infection, the WHO underlined to need to use inexpensive non-invasive approaches to assess the extent of liver fibrosis in resource-limited settings. However, they also recommend to use alternative approaches such as transient elastography wherever available. Although being initially expensive, the cost of transient elastography compared to alternative non-invasive biological tests for the evaluation of liver fibrosis remains to be assessed in the long term. Finally, the cross-sectional nature of the study limits our ability to draw any causal relation between liver fibrosis and associated factors. Nevertheless, several factors including HIV infection or the use of herbal medicines have been previously incriminated in previous cross-sectional studies. A longitudinal assessment of the impact of these factors on liver fibrosis will ultimately be needed.

## Conclusion

Infection with HIV and HBV were high in inmates incarcerated in the state prisons of Lome and Dakar. Although being relatively infrequent, severe liver fibrosis was clearly and independently associated with both chronic hepatitis infections and HIV infection. In a context of increasing availability of efficient antiviral therapies for HIV and chronic hepatitis infections, appropriate monitoring strategies for liver disease need to be explored in vulnerable populations, including prison inmates. The association between traditional medicine use and liver fibrosis highlights the need to provide better documentation of etiologic factors leading to liver damages in sub-Saharan Africa.

## Abbreviations

ALT, Alanine aminotransferase; AST, Aspartate aminotransferase; AUDIT, Alcohol Use Disorder Identification Test; BMI, Body Mass Index; 95 % CI, 95 % Confidence Interval; HBV, Hepatitis B Virus; HCV, Hepatitis C Virus; HIV, Human Immunodeficiency Virus; OR, Odds Ratio; USA, United States of America; WHO, World Health Organization
